# Natural brain state change with E/I balance shifting toward inhibition is associated with vigilance impairment

**DOI:** 10.1016/j.isci.2023.107963

**Published:** 2023-09-20

**Authors:** Binghao Yang, Haoran Zhang, Tianzi Jiang, Shan Yu

**Affiliations:** 1 Brainnetome Center, Laboratory of Brain Atlas and Brain-inspired Intelligence, Institute of Automation, Chinese Academy of Sciences, Beijing 100190, China; 2 School of Future Technology, University of Chinese Academy of Sciences, Beijing 101408, China; 3 School of Artificial Intelligence, University of Chinese Academy of Sciences, Beijing 101408, China; 4 Research Center for Augmented Intelligence, Zhejiang Lab, Hangzhou 311121, China

**Keywords:** Physiology, Neuroscience, Behavioral neuroscience, Sensory neuroscience, Cognitive neuroscience

## Abstract

The delicate balance between cortical excitation and inhibition (E/I) plays a pivotal role in brain state changes. While previous studies have associated cortical hyperexcitability with brain state changes induced by sleep deprivation, whether cortical hypoexcitability is also linked to brain state changes and, if so, how it could affect cognitive performance remain unknown. Here, we address these questions by examining the brain state change occurring after meals, i.e., postprandial somnolence, and comparing it with that induced by sleep deprivation. By analyzing features representing network excitability based on electroencephalogram (EEG) signals, we confirmed cortical hyperexcitability under sleep deprivation but revealed hypoexcitability under postprandial somnolence. In addition, we found that both sleep deprivation and postprandial somnolence adversely affected the level of vigilance. These results indicate that cortical E/I balance toward inhibition is associated with brain state changes, and deviation from the balanced state, regardless of its direction, could impair cognitive performance.

## Highlights

• Brain state change can be induced by postprandial somnolence or sleep deprivation
• Both types of brain state change lowered the level of vigilance
• Postprandial somnolence is hypoexcitatory while sleep deprivation is hyperexcitatory

## Graphical abstract

**Figure:**
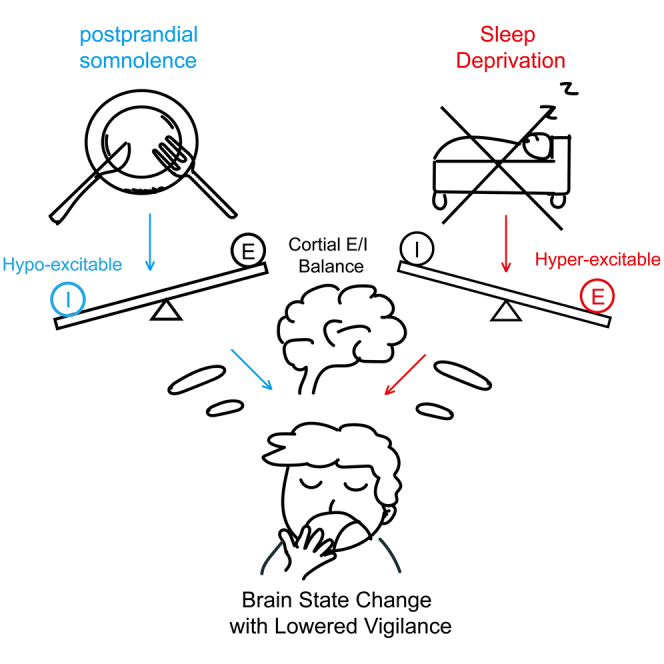

## Introduction

The natural change of the brain state, such as sleep,^1^^,^^2^^,^^3^ wakefulness,^4^ and drowsiness,^5^ is an everyday experience for us and affects the cognitive functions. Studying the mechanism beneath the brain state change is an important topic to understand the brain dynamics and its relation with cognition.^6^ Cortical excitation/inhibition balance (E/I balance) has been suggested as an useful framework to study the organization of cortical activities and evaluate brain functions.^7^^,^^8^^,^^9^^,^^10^^,^^11^ The E/I balance emerges and can be measured at multiple levels. At the level of the single neuron, the E/I balance can be manifested as highly synchronized and correlated synaptic excitatory and inhibitory currents.^12^ At the network level, the state associated with balanced E/I can be diagnosed by the scale-free characteristics of network activity, such as avalanche dynamics reflected by power-law distribution^13^^,^^14^^,^^15^^,^^16^ and long-range temporal correlation (LRTC) measured with the detrended fluctuation analysis (DFA).^17^^,^^18^^,^^19^

The balanced E/I in the neuronal network is associated with the optimal brain state with high efficiency of information coding,^20^ transmission,^21^ and processing,^13^^,^^14^^,^^15^ contributing to better cognitive performance, such as working memory^16^ and decision-making.^22^ Importantly, it is known that the shifting of E/I balance is related to the change of the brain state. In natural conditions, the E/I balance can be regulated by the circadian processes^23^^,^^24^^,^^25^ and previous studies indicated that the E/I balance shifting toward excitation is associated with the brain state change induced by the sleep deprivation,^26^^,^^27^^,^^28^^,^^29^ which impairs cognitive function significantly.^30^^,^^31^

Despite evidence that the E/I balance shifting toward excitation is associated with the brain state change caused by the sleep deprivation, whether the E/I balance shifting toward inhibition in the day time can also be linked to the natural brain state change and its influence on the cognitive functions remains unclear. In order to address these questions, we investigated another form of the brain state change that commonly occurs after meal called postprandial somnolence,^32^^,^^33^^,^^34^ which is not mediated by the circadian rhythm. Specifically, we measured the changes in the vigilance level due to the postprandial somnolence by a psychomotor vigilance task (PVT^35^), while the associated changes in cortical dynamics were studied by analyzing EEG-based network E/I balance parameters. Both behavioral and electrophysiological results were then compared with the condition of the sleep deprivation. We found that these two types of drowsiness states showed similar behavior performance deficits but the postprandial somnolence was associated with the E/I balance shifting toward inhibition, which was opposite to that after the sleep deprivation. Our results provide evidence supporting the existence of E/I balance shifting toward inhibition under the natural brain state change that impairs the cognitive performance, providing a more complete understanding about the interplay between cortical E/I balance, brain state changes, and cognitive performance, which could inform potential interventions to optimize brain states and cognitive abilities.

## Result

### Behavioral analysis

**Figure 1:**
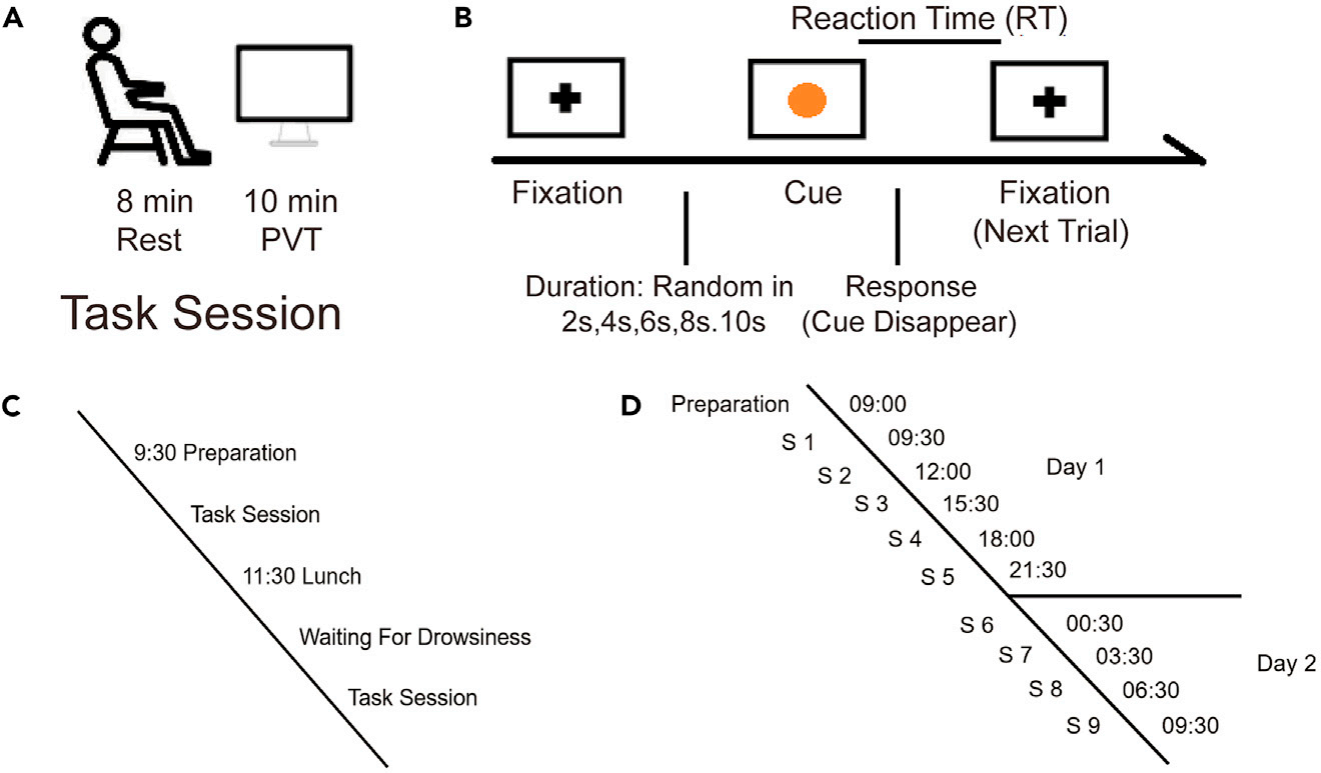
Experiment procedure (A and B) (A) Task session, (B) Illustration of a PVT trial. (C) The experiment procedure of the postprandial somnolence. (D) The experiment procedure of the sleep deprivation.

The experimental procedure of the postprandial somnolence is shown in Figure 1C. Participants arrived at the laboratory at about 9:30 a.m. and finished a task session after EEG preparation. The task session contained an 8-min resting time and a 10-min PVT. Participants had lunch at 11:30 a.m., as shown in Figure 1A and after that took another task session when they reported being drowsy. The experimental procedure of the sleep deprivation is shown in Figure 1D. Participants arrived at the laboratory at about 9:00 a.m. on Day 1 and completed 9 task sessions the same as the task session in the experiment of postprandial somnolence in a total 22-h course of sleep deprivation. A PVT trial is shown in Figure 1B. In brief, a fixation was presented at the center of the screen. Then the fixation disappeared and a yellow circular cue occurred. Participants were asked to press any key on the keyboard as soon as possible for response when noticing the cue occurrence. Reaction time (RT) for each trial was collected. For details of the experimental set up and procedures, please see the STAR methods section.

**Figure 2:**
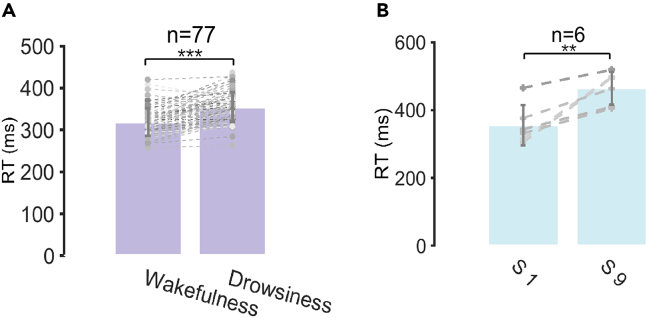
Behavior analysis (A) RT change associated with the postprandial somnolence (n = 77). (B) RT change associated with the sleep deprivation (n = 6). S1: the first session in the sleep deprivation course, with the subjects in the wakefulness state; S9: the last session in the sleep deprivation course, with the subjects in the drowsiest state. ∗∗p < 0.01, ∗∗∗p < 0.001; Two-sided paired t-test. Error bars represent the standard deviation.

Vigilance performance changes under both drowsiness situations were examined by analyzing the RT in the PVT task, shown in Figure 2. We found that the RT was significantly longer under the postprandial somnolence condition than the wakefulness condition (Figure 2A, p < 0.001, Cohen’s d = 1.1805). Similarly, at the last session (S9) during the sleep deprivation, the RT was significantly longer (Figure 2B, p = 0.0073, Cohen’s d = 1.7793) compared with the wakefulness condition at the first session (S1). In Figure S3 we show the distribution of RTs in conditions of wakefulness, the postprandial somnolence and the sleep deprivation. Overall, the increases in RT indicated the decline of the vigilance level under both drowsiness states.

### E/I balance analysis of the postprandial somnolence group

**Figure 3:**
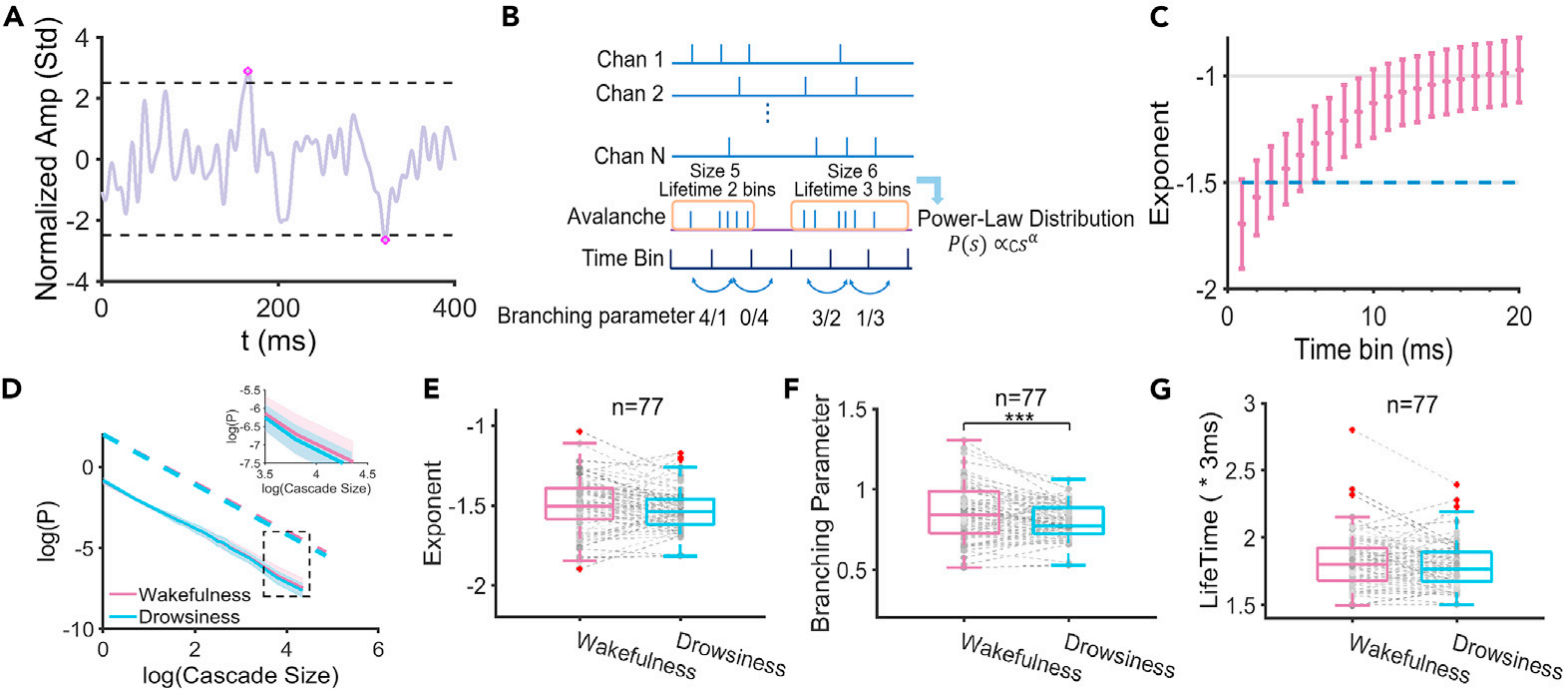
Neuronal avalanche analysis in the postprandial somnolence group (A) Illustration of the event detection. Positive and negative events beyond the threshold of ±2.5SD were identified in each channel. (B) Neuronal avalanche analysis. The size of an avalanche was defined as the sum of event numbers in it. The branching parameter was defined as the quotient of events number in adjacent time bins. The lifetime was defined as the duration of an avalanche. (C) The avalanche exponent with different time bin lengths under the wakefulness condition. The time bin of 3-ms was chosen for the subsequent analysis. (D) Power-law distribution of the wakefulness and the postprandial somnolence conditions. The avalanche exponent was estimated with the whole range of x axis of the double-log plot, from 0 to 4.3594 (e^4.3594^ = 78.2) under the wakefulness condition and from 0 to 4.3466 (e^4.3466^ = 77.2) under the drowsiness condition, respectively with considering all data points for more precise and less biased estimation. (E) Changes in the avalanche exponent (n = 77). (F) Changes in the branching parameter (n = 77). (G) Changes in the avalanche lifetime (n = 77). The solid line represents the mean value, and the shadow represents the standard deviation. Gray dots represent individual data points. ∗∗∗p < 0.001; Two-sided paired t-test.

The result of neuronal avalanche analysis under the postprandial somnolence is shown in Figure 3. Firstly the events were identified as super-threshold signals for individual channels (Figure 3A) with a specific time bin, then the avalanches were defined as a series of continuous events cascades unfolding across all channels (Figure 3B). The size and lifetime of an avalanche were defined as the total number of events within the avalanche and the total duration of it, respectively. In the critical dynamics, the avalanche size obeys the power-law distribution $P\left(s\right)\propto cs^{\alpha }$ with the exponent α close to −3/2, which can be fitted as a straight line under the double-logarithmic coordinates.^36^ Importantly, for avalanche dynamics, the branching parameter *σ*, which is defined as the average value among all quotients of the event number of the latter time bin divided by the former time bin, measures the excitability of the network.^37^ The increased/decreased avalanche exponent and branching parameter suggests the network dynamics shifting toward excitation/inhibition.

We found that the avalanche exponent of the wakefulness condition was approximately −3/2 with a 3-ms time bin (Figure 3C), so this time bin length was adopted for further analysis in the postprandial somnolence group. In our results, the shape of the avalanche size distribution, including the estimated avalanche exponent, across all subjects under the wakefulness and the postprandial somnolence condition didn’t show a significant difference (Figures 3D and 3E, p = 0.1251, Cohen’s d = 0.1768). However, the branching parameter was significantly lower in the postprandial somnolence compared to the wakefulness condition (Figure 3F, p < 0.001, Cohen’s d = 0.4033). As a supplement, the kappa index showed near-significant decrease from the wakefulness to the postprandial somnolence (Figure S4A, p = 0.0991, Cohen’s d = 0.1903). There was no significant change in the avalanche lifetime between these two conditions (Figure 3G, p = 0.1790, Cohen’s d = 0.1546), so the decline of the branching parameter was not induced by the reduction of the avalanche lifetime but mainly represented the reduced activity propagation in the postprandial somnolence.

**Figure 4:**
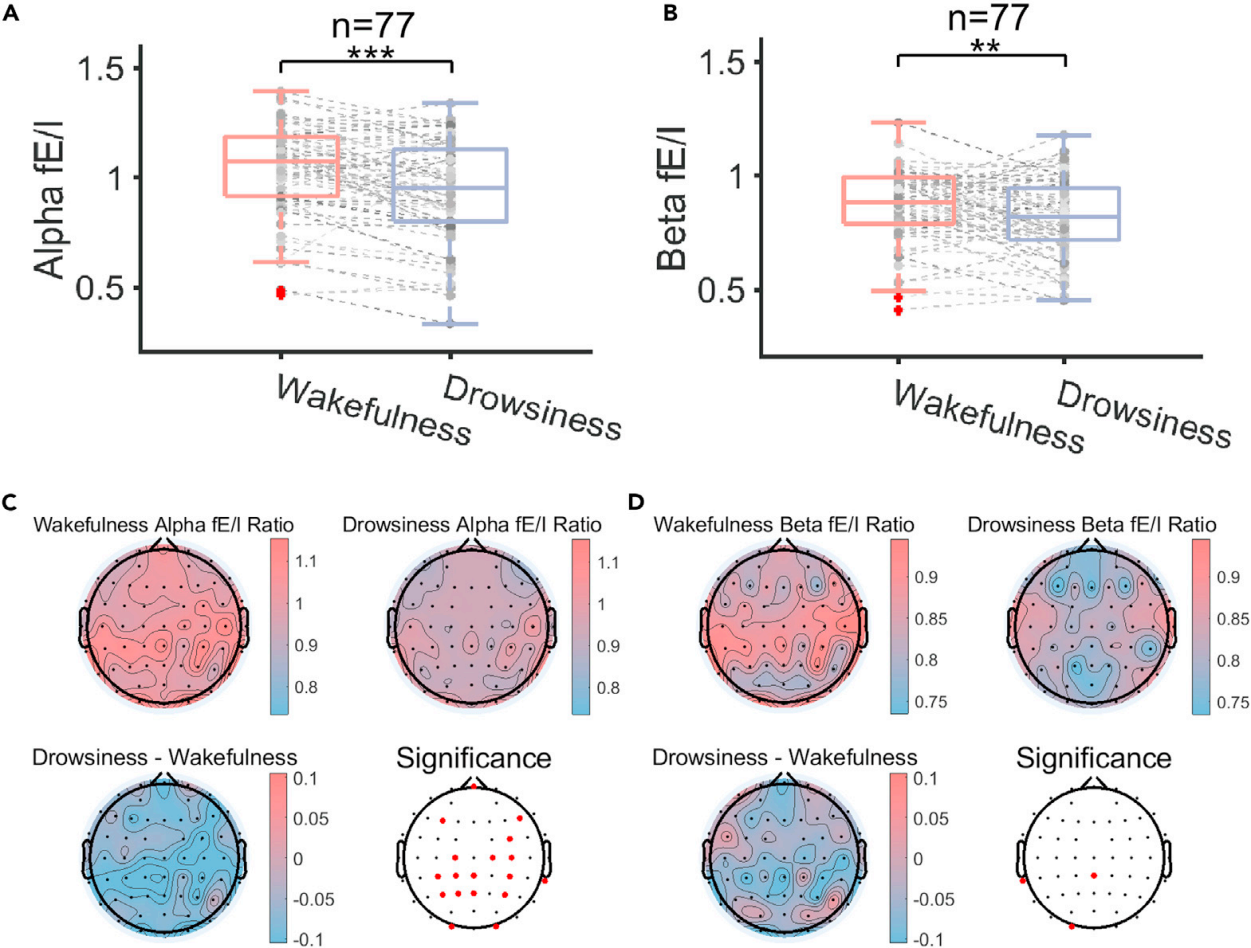
The fE/I ratio analysis in the postprandial somnolence group (A) Changes in the alpha band fE/I ratio (n = 77). (B) Changes in the beta band fE/I ratio change (n = 77). ∗∗p < 0.01, ∗∗∗p < 0.001; Two-sided paired t-test. (C) Spatial distribution of the alpha fE/I ratio. Top-left: the spatial distribution of the alpha fE/I ratio under the wakefulness condition. Top-right: the spatial distribution of the alpha fE/I ratio under the postprandial somnolence condition. Bottom-left: the spatial distribution of the difference of the alpha fE/I ratio between the wakefulness and the postprandial somnolence condition. Bottom-right: the spatial distribution of the significance of the alpha fE/I ratio change at individual channel level between the wakefulness and the postprandial somnolence condition. The red dots label significant change at individual channels. (D) Spatial distribution of the beta fE/I ratio. The panels are arranged similarly to (C). Gray dots represent individual data points. Two-sided paired t-test, spatial significance after Bonferroni correction.

The avalanche analysis can be applied to measure the E/I balance at the network level. To reconfirm the results at network level and to measure the state of E/I balance for individual channels, we used another method called fE/I ratio that measures the relationship between the LRTC and the amplitude of single-channel EEG signals.^38^ We tested all frequency bands of the fE/I ratio and found significant changes in the alpha band (8–12 Hz), $k_{\alpha }$, and the beta band (12–30 Hz), $k_{\beta }$. The increased/decreased fE/I ratio suggests the network shifting toward excitation/inhibition. At the whole-brain level, we found that both the whole-brain alpha band (Figure 4A, p < 0.001, Cohen’s d = 0.5609) and beta band fE/I ratio (Figure 4B, p = 0.0063, Cohen’s d = 0.3205) were significantly lower under the postprandial somnolence compared with the wakefulness condition. At the level of individual channels, the channels showing significantly lowered fE/I ratio in both alpha and beta bands from the wakefulness to the postprandial somnolence condition mainly distributed at the parietal lobe (p < 0.05, Bonferroni-Corrected, Figures 4C and 4D). Overall, the results suggested that the E/I balance of the cortical network evolved toward inhibition from the wakefulness to the postprandial somnolence, and these changes could be detected at the whole-brain level and the level of individual recording channels.

### E/I balance analysis of the sleep deprivation group

**Figure 5:**
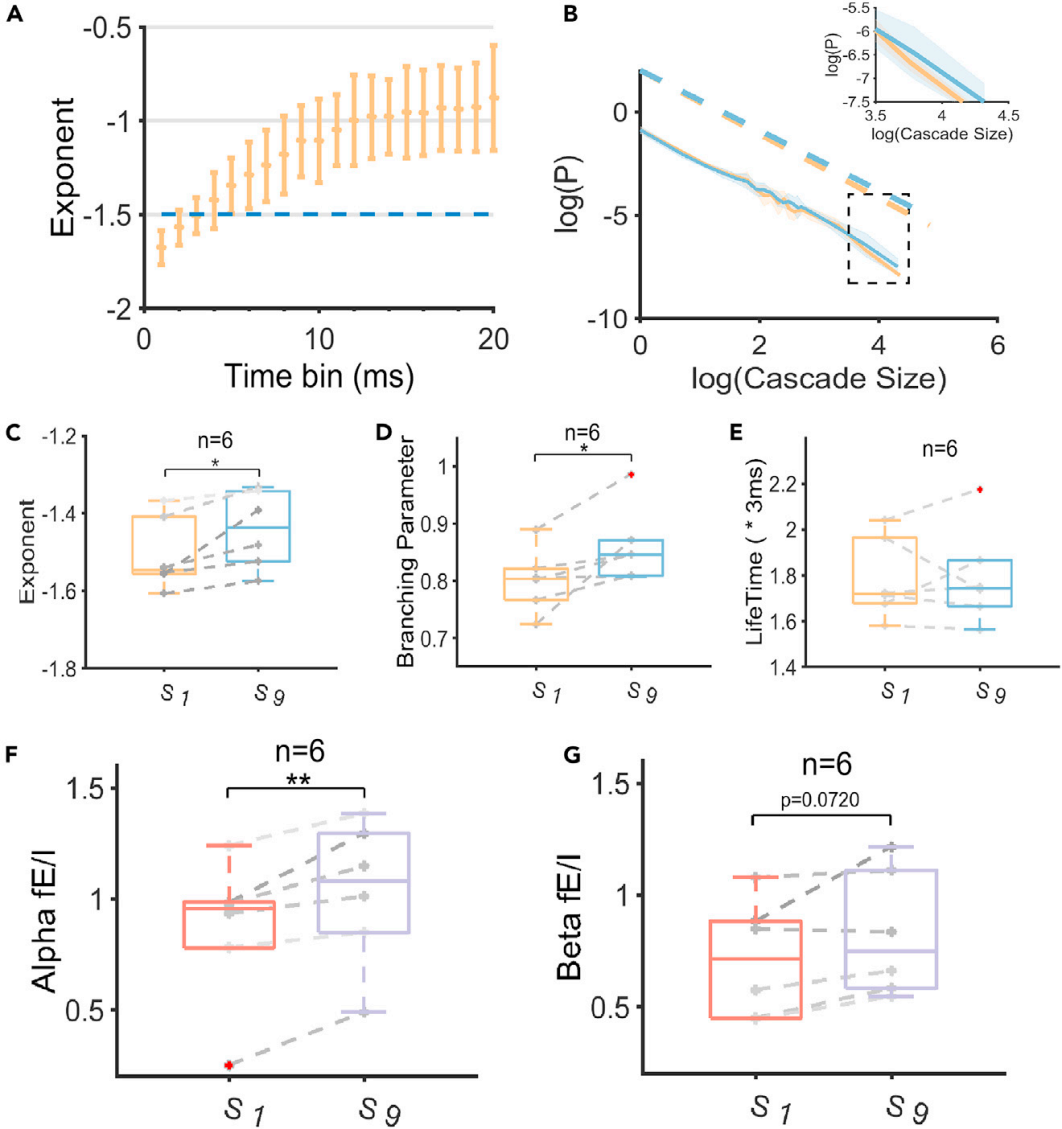
E/I balance analysis in the sleep deprivation group (A) The avalanche exponent with different time bin lengths under the wakefulness condition. The time bin of 3-ms was chosen for the subsequent analysis. (B) Power-law distribution of the wakefulness and the sleep deprivation condition. The avalanche exponent was estimated with the whole range of x axis of the double-log plot, from 0 to 4.3555 (e^4.3555^ = 78) under the S1 condition and from 0 to 4.3182 (e^4.3182^ = 75) under the S9 condition, respectively with considering all data points for more precise and less biased estimation. (C) Changes in the avalanche exponent (n = 6). (D) Changes in the branching parameter (n = 6). (E) Changes in the avalanche lifetime (n = 6). (F) Changes in the alpha band fE/I ratio (n = 6). (G) Changes in the beta band fE/I ratio (n = 6). S1: the first session in the sleep deprivation course, with the subjects in the wakefulness state; S9: the last session in the sleep deprivation course, with the subjects in the drowsiest state. The solid line represents the mean value, and the shadow represents the standard deviation. Gray dots represent individual data points. ∗p < 0.05, ∗∗p < 0.01; Two-sided paired t-test.

The result of the E/I analysis of the sleep deprivation group is shown in Figure 5. Similarly, we found that the avalanche exponent of the wakefulness condition was approximately −3/2 with a 3-ms time bin (Figure 5A), so this time bin length was used for further analysis in the sleep deprivation group. The neuronal avalanche exponent in the wakefulness condition at the first session of the sleep deprivation (S1) was significantly higher than the drowsiness condition at the last session of the sleep deprivation (S9) (Figures 5B and 5C, p = 0.0313, Cohen’s d = 1.2113). In addition, the branching parameter was significantly higher in S9 compared to S1 (Figure 5D, p = 0.0398 Cohen’s d = 1.1271) As a supplement, the kappa index showed significant increase from S1 to S9 (Figure S4B, p = 0.0310, Cohen’s d = 1.2148). The avalanche lifetime between S1 and S9 didn’t show a significant change (Figure 5E, p = 0.8816, Cohen’s d = 0.0640). These results illustrated the enhanced activity propagation under the sleep deprivation. In addition, the alpha band fE/I ratio in S9 was significantly higher than that in S1 (Figure 5F, p = 0.0073, Cohen’s d = 1.7822). The beta band fE/I showed a near-significant increase from S1 to S9 (Figure 5G, p = 0.0720, Cohen’s d = 0.9286). Taken together, the cortical network turned toward excitation under the sleep deprivation condition compared with the wakefulness condition.

### Gamma power analysis

**Figure 6:**
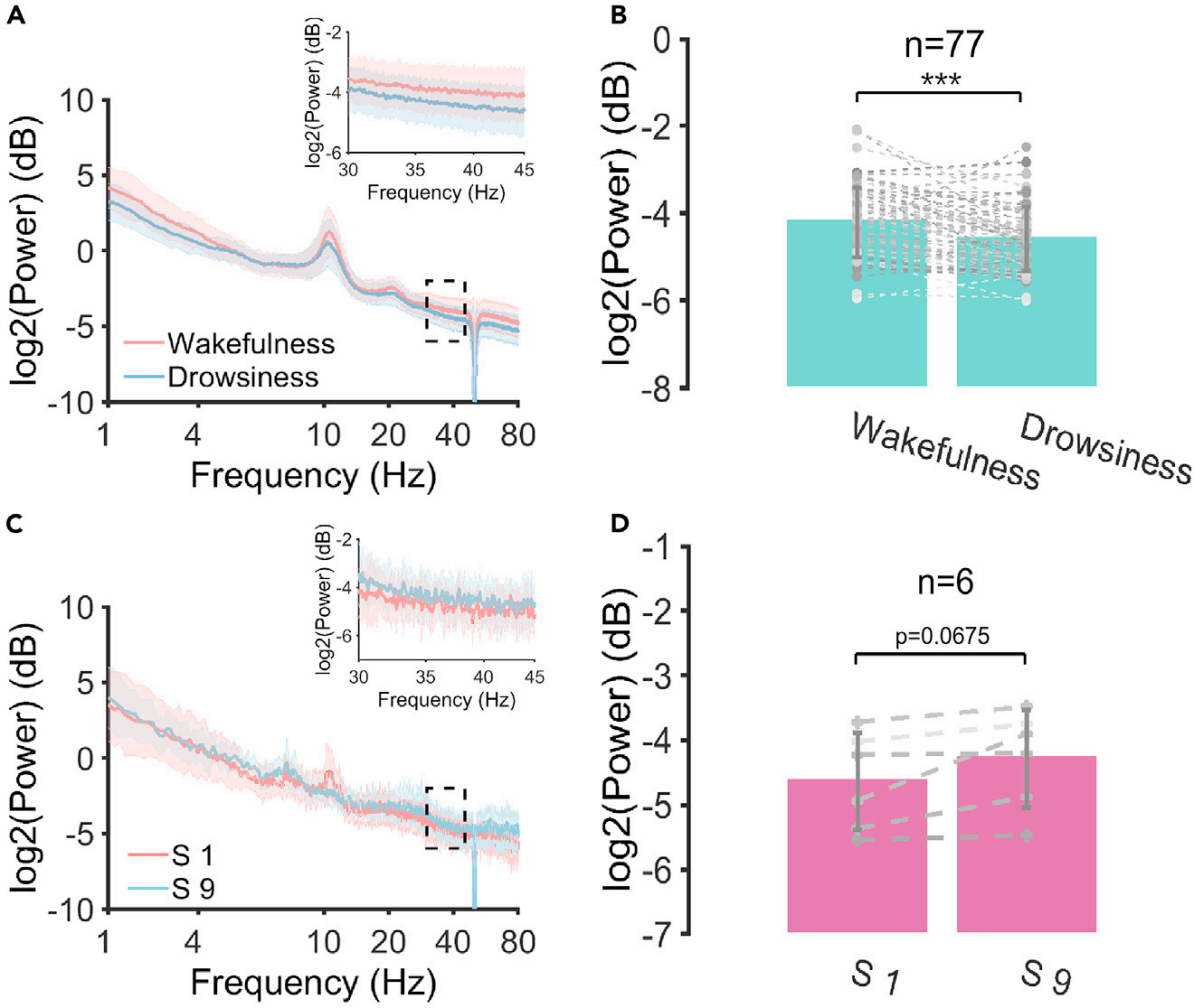
Frontal gamma power change (A) Power spectrum associated with the postprandial somnolence group. (B) Changes in the gamma power associated with the postprandial somnolence group (n = 77). (C) Power spectrum associated with the sleep deprivation group. (D) Changes in the gamma power associated with the sleep deprivation group (n = 6). The solid line represents the mean value, and the shadow represents the standard deviation. ∗∗∗p < 0.001; Two-sided paired t-test. Error bars represent the standard deviation.

The gamma band power of EEG signals, as a widely used measure in many studies regarding brain state changes, exhibits a specific relationship with the E/I balance,^39^ with enhanced gamma power indicating shifting of the E/I balance toward excitation. Here, the frontal gamma power γ (30–45 Hz) was analyzed under different drowsiness types. The frontal gamma power was calculated by averaging the gamma power among all frontal-located channels. As shown in Figure 6, gamma power showed a decline under the postprandial somnolence condition compared with the wakefulness condition (Figure 6B, p < 0.001, Cohen’s d = 0.6046). This result indicated the association between the prolonged RT and the changes in the gamma power. In contrast, the frontal gamma power showed a near-significant increase in S9 compared with S1 (Figure 6D, p = 0.0675, Cohen’s d = 0.9496).

### Modeling analysis

**Figure 7:**
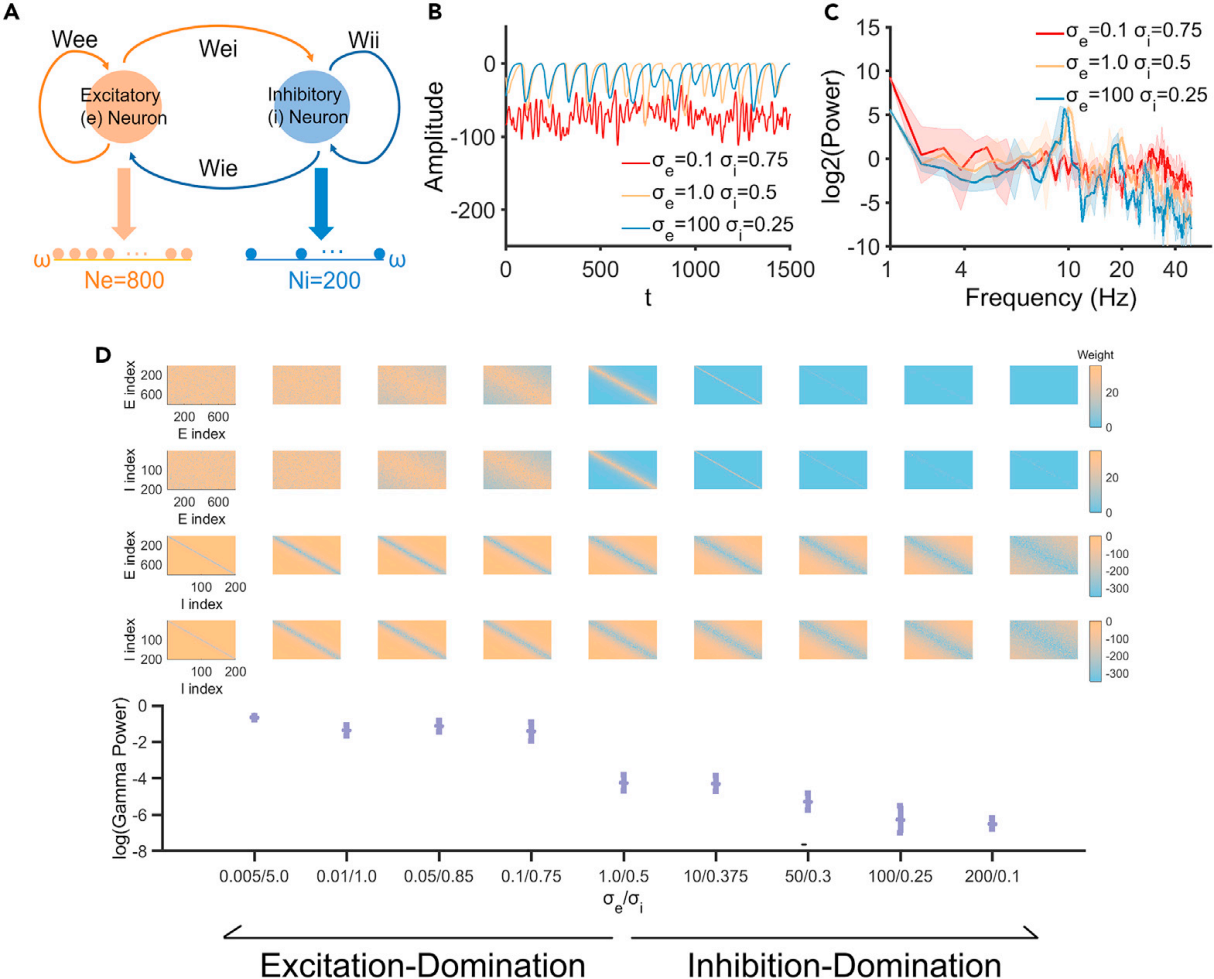
Modeling results (A) The structure of the network model. ***W***_***ee***_: the excitatory synaptic connections within excitatory neurons. ***W***_***ei***_: the excitatory synaptic connections from excitatory neurons to inhibitory neurons. ***W***_***ie***_: the inhibitory synaptic connections from inhibitory neurons to excitatory neurons. ***W***_***ii***_: the inhibitory synaptic connections within inhibitory neurons. The excitatory and inhibitory neurons were uniformly arranged on two separate one-dimensional spaces with the length of ω. (B) Output waveforms of the network with the initial parameter (*σ*_*e*_ = 1.0, *σ*_*i*_ = 0.5), the inhibition-dominant parameter (*σ*_*e*_ = 100, *σ*_*i*_ = 0.25) and the excitation-dominant parameter (*σ*_*e*_ = 0.1, *σ*_*i*_ = 0.75). (C) Power spectrum of the network output with different E/I parameters. (D) The model weights and gamma power changes with different excitation and inhibition parameters. E index represents the ordinal number of the excitatory neuron. I index represents the ordinal number of the inhibitory neuron. The first row of colored squares: the excitatory synaptic connection weights of ***W***_***ee***_. The second row of colored squares: the excitatory synaptic connection weights of ***W***_***ei .***_The third row of colored squares: the inhibitory synaptic connection weights of ***W***_***ie .***_ The fourth row of colored squares: the inhibitory synaptic connection weights of ***W***_***ii .***_ When the network enters the excitation-dominant state, the excitatory weights increase (more positive) as well as the inhibitory weights increase (less negative). When the network enters the inhibition-dominant state, the excitatory weights decrease (less positive) as well as the inhibitory weights decrease (more negative). The orange color represents high value and the blue color represents low value of weights. The solid line represents the mean value, and the shadow represents the standard deviation.

A computational model was built to further explore the relationship between the E/I balance and the gamma power. The structure of the network model is shown in Figure 7A. ***W***_***ee***_ and ***W***_***ei***_ represent the excitatory synaptic connection weights of the network controlled by the excitation parameter $\sigma_{e}^{2}$, and ***W***_***ie***_ and ***W***_***ii***_ represent the inhibitory synaptic connection weights of the network controlled by the inhibition parameter $\sigma_{i}^{2}$. The E/I balance change relative to the initial state of the network could be manipulated by adjusting $\sigma_{e}^{2}$ and $\sigma_{i}^{2}$ i.e., when increasing $\sigma_{e}^{2}$ and decreasing $\sigma_{i}^{2}$, the network shifted toward inhibition, and when increasing $\sigma_{i}^{2}$ and decreasing $\sigma_{e}^{2}$, the network shifted toward excitation. Figure 7 shows the model output waveforms (Figure 7B), their spectrum analysis (Figure 7C) and the gamma power change (Figure 7D) with different model E/I parameters $\sigma_{e}^{2}$ and $\sigma_{i}^{2}$. Under the initial parameter, the model showed spontaneous ∼10 Hz activity reflected on the power spectrum. When adjusting $\sigma_{e}^{2}$ and $\sigma_{i}^{2}$, the E/I balance changed and the gamma power of the model output under different parameter combinations was calculated. When increasing $\sigma_{e}^{2}$ and decreasing $\sigma_{i}^{2}$, the network entered an inhibition-dominant state and the gamma power decreased, similar to the EEG result of the postprandial somnolence state. When increasing $\sigma_{i}^{2}$ and decreasing $\sigma_{e}^{2}$, the network entered an excitation-dominant state and the gamma power increased, similar to the EEG result of the sleep deprivation condition. Through this model, we verify the relationship between the gamma power and the E/I balance: the E/I balance shifted toward inhibition is associated with the decreasing gamma power, and the E/I balance shifted toward excitation is associated with the increasing gamma power.

## Discussion

In the day time, the natural brain state is not invariant but shows heterogeneity and this change can be described by the cortical E/I balance disturbance. Previous studies have indicated the brain state change accompanied with the E/I balance shift toward excitation under sleep deprivation. The LRTC characteristic of the neuronal activity qualified by the DFA exponent declined during sleep deprivation, which indicated the disruption of critical dynamics.^29^ Neuronal avalanche analysis indicated an increased branching parameter and deviation from the critical dynamics, implying the E/I balance shifting toward a hyper-excitatory state after the sleep deprivation.^28^ Further, the amplitude of the transcranial magnetic stimulation (TMS) evoked potential increased during the sleep deprivation, confirming the enhanced cortical excitability, which could be restored by sleep.^26^^,^^27^ In line with these studies, we verified that the E/I balance shifted toward excitation under the sleep deprivation based on the EEG analyses.

But whether E/I balance shifting toward inhibition can be linked to the natural brain state change in the day time remains unknown. To answer this question, we studied a typical form of brain state change that mainly occurs after meal called postprandial somnolence. The postprandial somnolence is common among humans, acting as an increased sleep tendency after a meal.^40^ Previous EEG analysis indicated increased theta and alpha power accompanied with increased sleepiness after a meal.^41^ However, the changes in the E/I balance under the postprandial somnolence have not been clarified. Here, we found the E/I balance shifted toward inhibition under the postprandial somnolence, which was opposite to the sleep deprivation.

The maintenance of the E/I balance is essential for optimal brain function. Theoretical works indicated that the E/I balanced state gave rise to the stable firings across different levels of the brain network to keep spiking activity propagation with high fidelity and information transmission more reliable.^42^ *In vitro* experiments demonstrated information capacity and transmission were maximized within a balanced E/I.^21^ Moreover, the relation between perturbations in the E/I balance and cognitive impairments as well as neuropsychiatric disorders has been indicated. Specifically, autism,^38^^,^^43^^,^^44^ schizophrenia^45^^,^^46^ and Alzheimer’s disease^47^^,^^48^ were associated with E/I balance shifting toward excitation, while major depressive disorder was associated with E/I balance shifting toward inhibition,^49^ with both directions of deviation affecting cognition adversely. Except for neuropsychiatric disorders, we illustrated the relation between E/I balance shift and temporally cognitive impairment that naturally occurred in the day time. Because of the sensitivity of vigilance level to changes in the brain state,^30^^,^^35^ here, we tested the vigilance performance by PVT under both the postprandial somnolence and the sleep deprivation. Both the postprandial somnolence and the sleep deprivation induced an increase in RT, indicating the natural bidirectional E/I balance disturbance affected cognitive functions.

The E/I balance shift at the neuronal network level can be regulated by the cellular E/I ratio of neurons. In pyramidal cells of visual and prefrontal cortices as well as hippocampal CA1 on mice, synaptic inhibition and excitation showed oscillation but with different change direction over the 24-h light/dark cycle, demonstrating the large change of E/I balance across a day.^23^ Previous *in vitro* and *in vivo* animal experiments have illustrated the increase of synaptic potential under the sleep deprivation was associated with an increase of AMPA receptor density in synapses. This change was followed by a related increase in the amplitude and frequency of excitatory postsynaptic currents (EPSC) and an increase of firing rate, as well as enhanced synchronization of cortical neurons.^50^^,^^51^ During sleep deprivation, the accumulation of adenosine could act on adenosine A_2A_ receptors to mediate hyper-excitation in the basal ganglia, hippocampus and neocortex by enhancing glutamatergic synapses directly or altering glutamate transport.^52^^,^^53^^,^^54^ This cellular mechanisms provided an explanation to the macroscopic E/I balance shifting toward excitation after the sleep deprivation. But the cellular mechanism accounting for the E/I balance shifting toward inhibition under the postprandial somnolence is still an open question. The postprandial somnolence might be induced by the reduction of orexin release due to elevated blood glucose after a meal,^33^ and future studies are needed to investigate the influence of orexin reduction on the cortical E/I balance.

When measuring E/I balance at the network level, TMS evoked potential is a common method. Applying a TMS pulse delivered an induced electric field on the cortical surface and activated the cortex.^27^^,^^55^^,^^56^ The evoked cortical potential could be detected by EEG and the cortical excitability could be measured by the slope or the amplitude of the evoked potential.^26^^,^^27^^,^^57^ But this method only measured the excitability of a localized narrow range of cortical areas. Here, we took three methods measuring wide E/I balance based on the resting-state EEG recording: neuronal avalanche analysis, fE/I ratio and gamma power. The results among these methods were consistent to indicate the E/I balance shift. Neuronal avalanche analysis was based on EEG recorded in multiple sensors to measure the power-law characteristics of critical neuronal dynamics.^11^^,^^37^^,^^58^^,^^59^^,^^60^^,^^61^ The fE/I ratio could be calculated at the single-channel level and the span of the E/I balance shift could be described by the whole brain topographical distribution.^38^ Resting-state gamma power has been suggested to examine the E/I balance. The experimental study has found that gamma power was negatively correlated with GABA concentration and positively correlated with the glutamate/GABA ratio.^39^ Here, we built a computational model to further illustrate this relationship. When manipulating the E/I balance in the model network by adjusting the E/I weights, the output gamma power changed accordingly, that was, the gamma power changed along with the E/I balance shift. Specifically, the gamma power decreased with the E/I balance shifting toward inhibition but increased with the E/I balance shifting toward excitation, which was consistent with both previous empirical findings^39^^,^^62^ and our EEG-based results reported here.

Our results would be instrumental for selecting the proper intervening method that could modulate the E/I balance bidirectionally, such as theta burst stimulation (TBS), to alleviate the functional deficits caused by different types of drowsiness, which were important for many practical applications such as driving. TBS was designed as delivering TMS pluses with gamma band frequency at intervals with theta band frequency.^63^^,^^64^ TBS could be divided into two protocols: continuous TBS (cTBS), in which TBS was delivered in an uninterrupted train for typically 40 s, as well as intermittent TBS (TBS), in which 2 s TBS trains were repeated every 10 s for a totally 190 s.^63^ Different TBS protocol was indicated to have different mediation effects on E/I balance. Specifically, cTBS was commonly considered inhibitory while iTBS was regarded as excitatory.^63^^,^^65^ According to our results, iTBS and cTBS might be suited for alleviating functional deficits caused by postprandial somnolence and sleep deprivation, respectively. Future studies empirically investigating these effects would provide a critical test for the current conclusion.

### Limitations of the study

We would like to note a few limitations of the current study. First, we have a relatively small sample size for the sleep deprivation group (n = 6) compared with the postprandial somnolence group (n = 77). Second, the majority of the subjects were male (6/6 in the sleep deprivation group and 60/77 in the postprandial somnolence group). It needs future studies to verify our results of the sleep deprivation with larger sample size and more balanced sex ratio in subjects. Third, the computational model built in our study is only designed to describe the gamma power change under different E/I ratios. Future studies can build more comprehensive computational model to study more aspects of E/I ratio corresponding to changes in other electrophysiological parameters reported in our experiment.

## STAR★Methods

### Key resources table

**Table:** 

REAGENT or RESOURCE	SOURCE	IDENTIFIER
**Deposited data**		

Preprocessed study data	this paper	https://doi.org/10.6084/m9.figshare.23514234

**Software and algorithms**		

Analysis code	this paper	https://doi.org/10.6084/m9.figshare.23514246
MATLAB R2018a	MathWorks	http://www.mathworks.com
MATLAB EEGLAB	Delorme A et al., 2004^66^	https://sccn.ucsd.edu/eeglab/index.php
MATLAB Psychtoolbox3	Brainard, 1997^67^	psychtoolbox.org

**Other**		

Brain Products System	Brain Products GmbH	https://www.brainproducts.com/

### Resource availability

#### Lead contact

Further information and requests for resources and reagents should be directed to and will be fulfilled by the lead contact, Shan Yu (shan.yu@nlpr.ia.ac.cn).

#### Material availability

This study did not generated new unique reagents.

### Experimental model and study participant details

Seventy-seven Chinese participants (60 males and 17 females, mean age ±SE = 25.2 ± 2.4) were recruited for the experiment of the postprandial somnolence. All participants were right-handed, low-caffeine consumers, with normal or corrected-to-normal vision and without any neurological disorders. All participants had the habit of taking a nap after lunch and reported postprandial somnolence in the study. They were asked to take a good sleep before the day of the experiment, and not to use any psychotropic substances for one day before the experiment as well as during the whole experimental procedure.

Six participants (6 males, mean age ±SE = 24.7 ± 0.8) were recruited for the experiment of a nearly 22 h sleep deprivation. Participants were asked to maintain good rest and not to use any psychotropic substances for three days before the experiment. Other requirements of participants were the same as the group of the postprandial somnolence.

Both experiments were approved by the institution’s ethics committee. All participants signed the informed consent document.

### Method details

#### Experiment procedure

The experimental procedure of the postprandial somnolence was shown in Figure 1C. Participants arrived at the laboratory at about 9:30 a.m. After EEG recording preparation, participants finished a task session, containing an 8-min resting time, which consisted of alternating periods of 20 s eyes-open and 20 s eyes-closed with the requirement to look at the white fixation point at the center of the black screen when eyes opened, followed by 10-min PVT, as shown in Figure 1A. Participants had lunch at 11:30 and after that took another task session when they reported being drowsy. The experimental procedure of the sleep deprivation was shown in Figure 1D. Participants arrived at the laboratory at about 9:00 a.m. on Day 1. After preparation, participants completed 9 task sessions same as the task session in the experiment of postprandial somnolence at 9:30, 12:30. 15:30, 18:30, 21:30, 00:30 (Day 1), 03:30, 06:30 and 7:30 (Day 2) (S1 - S9). Meals were arranged at 11:30 and 17:30 on Day 1. During the break between two sessions, participants were allowed to play games, watch movies, study or take a short walk nearby the laboratory room.

#### PVT details

During the PVT, the room was dimly lit with one lamb. The computer screen (Alienware, resolution 1920∗1080, 24.5 inches, 240 Hz refresh rate) was placed in front of the participants about 50 cm and the height of the screen was adjusted to keep stimuli presented at the center of the participants’ straight sight. Figure 1B showed the diagram of a PVT trial. A black cross fixation was presented at the center of the screen. Then the fixation disappeared and a yellow circular cue with 20 pixels radius appeared at the same position. When noticing the cue, participants were asked to press any key on the keyboard to respond to the cue as soon as possible. After the response, the cue disappeared and another fixation point appeared for the next trial. The duration of the fixation was randomly chosen among 2, 4, 6, 8 and 10 s. Each PVT contained a total of 100 trials. Trials with different fixation duration were equal in number. The reaction time (RT) for each PVT trial was recorded. The background color of the screen was white throughout the PVT. The presentation of the stimuli was controlled by MATLAB (MathWorks, Natick, MA) using the Psychophysics Toolbox Version 3 (Brainard).^66^ EEG was recorded during each task session.

#### EEG recording and preprocessing

EEG data was recorded using 64 Ag/AgCl sintered ring electrodes EasyCap with BrainAmp DC amplifiers (Brain Products GmbH, Gliching, Germany). The position of electrodes was arranged according to the standard 10–20 system. AFz was used as the ground and the reference was placed on the apex of the nose. A vertical electrooculogram (EOG) was recorded to monitor eye movements and eye blinks. The EEG signals were amplified and digitized at a sampling rate of 5000 Hz (0.016–100 Hz bandpass filtering), with the impedance of each electrode below 20 kΩ. Data acquisition was controlled through Brain Vision Recorder (version 1.03, Brain Products GmbH, Gliching, Germany).

EEG preprocessing was performed in EEGLAB (version 14.1.1)^67^ installed in MATLAB (MathWorks, Natick, MA). Raw EEG data was channel-located to the standard electrode location in the software and changed sampling rate to 1000 Hz. One electrode was used for recording the electrooculogram, and 4 other electrodes (FT9, FT10, TP9, TP10) distributed at the boundary of the EEG cap were excluded from analysis, and a total of 59 channels of the EEG data were used. Then the data was 1–80 Hz band-pass filtered offline followed by a notch filter at 50 Hz using 3 order zero-phase non-causal Butterworth filter. Resting-state data was divided into 20 s according to the eyes-open or eyes-closed condition. Segments with obvious movement artifacts were excluded. Then data segments were corrected for baseline drifting, and the parts with eye movements and blinks were removed by using independent component analysis (ICA). Only the middle 15 s of the resting period data segments with eyes closed were considered for further analysis.

#### Behavior analysis

The reaction time of each PVT session in both experiments was collected. Trials with too long or too short RT may not reflect the true response to the stimulus, so RT analysis considered trials with 200 ms < RT < 500 ms in the experiment of the postprandial somnolence, and trials with 200 ms < RT < 1000 ms in the experiment of the sleep deprivation, respectively.

#### Gamma power

For the resting state closed-eye EEG data, frontal channels (Fp1, Fp2, Fpz, AF7, AF3, AF4, AF8, F7, F5, F3, F1, F2, F4, F6, F8) were selected for calculating power spectrum and the gamma-band (30–45 Hz) power using Fast-Fourier Transform (FFT). Then the gamma power γ of the frontal lobe was calculated as the average among the gamma power of the frontal channels.

#### Neuronal avalanche analysis

According to the scale-free characteristic of the brain cortical network, the spontaneous activity propagation process could be described as the neuronal avalanches, which represented the criticality of network dynamics and indicated the E/I balance of the cortex. Here we took the method of neural avalanche analysis on resting MEG (Magnetoencephalography)^36^ to calculate the avalanche exponent, branching parameter and avalanche lifetime in our resting state closed-eye EEG data to measure the E/I balance, which were elaborated below. The neural avalanche analysis procedure was shown in Figure 3.

Specifically, data segments of individual subjects were concatenated. Then the single channel data were z-normalized by subtracting its mean and dividing by its standard deviation (SD) as well as detrended by the linear fluctuation. Positive and negative events beyond a threshold of each channel data were identified. In order to determine the threshold, we calculated the z-normalized signal amplitude distribution across all channels of all individuals, their grand average and the best fit Gaussian distribution. The separation of the grand average and the fitting Gaussian distribution determined the threshold. As displayed in Figure S1, both the wakefulness and the drowsiness condition under the postprandial somnolence and the sleep deprivation group showed that the grand average and the Gaussian fit started deviating from one another around ±2.5 SD, so the threshold was taken as ± 2.5 SD of each channel (Figure 3A).

After the determination of events, the following calculation process was applied to identify neural avalanches (Figure 3B). First, a time bin length should be determined. Then the sum of the number of events among all channels in each time bin was calculated. An avalanche, or a cascade, began at a time bin with at least one event and ended at a time bin without any event. Avalanche lifetime was defined as the total duration of avalanches. The sum number of events occurring in this avalanche was defined as the avalanche size. Previous studies suggested that at the critical state with balanced E/I, the avalanche size of all avalanches obeyed the power-law distribution $P\left(s\right)\propto cs^{\alpha }$, with exponent *α* close to −3/2. The *α* was estimated by the best-fitting straight line in the double-logarithmic coordinate here. In addition to the avalanche exponent, we also calculated the branching parameter to estimate the E/I ratio. The ratio *k* was defined as the quotient of the event number in the latter time bin divided by the event number in the former time bin for each bin pair in all avalanches. The quotient was 0 for the last bin in each avalanche. The branching parameter σ for individual subjects was calculated as averaged *k* across all bin pairs.

(Equation 1) $$\sigma =\frac{1}{N_{pairs}}\sum_{1}^{N_{pairs}}k=\frac{1}{N_{pairs}}\sum_{1}^{N_{pairs}}\frac{n_{latter\;time\;bin}}{n_{former\;time\;bin}}$$

The increased/decreased avalanche exponent and branching parameter suggested the network dynamics shifting toward excitation/inhibition from the wakefulness to the drowsiness condition. Further we tested the kappa index, *κ*, which is introduced to measure the deviation between the experimental cluster size cumulative density function (CDF) of the avalanche size, F(β), and the theoretical reference CDF, $F^{NA}\left(\beta \right)$,

(Equation 2) $$\kappa =1+\frac{1}{m}\sum_{k=1}^{m}\left(F^{NA}\left(\beta_{k}\right)-F\left(\beta_{k}\right)\right)$$

where $\beta_{k}$ indicates *m* = 10 avalanche sizes logarithmically spaced between the minimum and maximum avalanche size, as described in.^60^

Here we took the time bin length within which the wakefulness condition performed criticality i.e., the avalanche exponent was −3/2. The avalanche exponent change accompanied with the time bin length change was shown in Figure 3C for the wakefulness condition of the postprandial somnolence group, and shown in Figure 5A for the wakefulness condition of the sleep deprivation group. Both of them illustrated the avalanche exponent of the wakefulness condition was nearly −3/2 at a 3-ms time bin, so this time bin was considered for further analysis.

#### fE/I ratio analysis

Another method based on the relationship between the LRTC and the amplitude to quantify the E/I from neuronal oscillation was adopted here for the E/I balance analysis.^38^ In detail, the channel data was filtered, the amplitude envelope *A* was extracted and the average-removed cumulative sum of the amplitude envelope, as the signal profile *S*, was calculated as follows:

(Equation 3) $$S\left(t\right)=\sum_{k=1}^{t}\left(A\left(k\right)-\left[A\right]\right)$$

where [*A*] represented the mean amplitude of *A*. Then *S* was split into segments with overlapping windows. The segment in each window was normalized by dividing the mean value of the envelope amplitude of that window and then detrended. The standard deviation of each segment was calculated as the normalized fluctuation function *nF(t)*. Finally, the correlation coefficient $r_{amp,nF\left(t\right)}$ between the amplitude and the *nF(t)* among all segments was determined and fE/I was defined as:

(Equation 4) $$fE/I=1-r_{amp,nF\left(t\right)}$$

The calculation process was shown in Figure S2. Here the window length was 5 s with 80% overlapping. The increased/decreased fE/I ratio suggested the network dynamics shifting toward excitation/inhibition from the wakefulness to the drowsiness condition. We tested all frequency bands of the fE/I ratio and found significant changes in the fE/I ratio in the alpha band $k_{\alpha }$ (8–12 Hz) and beta band $k_{\beta }$ (12–30 Hz).

#### Modeling

A network model was built based on the model^68^ that was originally proposed to study spontaneous intrinsic alpha (∼10 Hz) oscillation. It contained the excitatory neurons (*e*) and inhibitory neurons (*i*) with the ratio 4:1. Spike train of each neuron ($X_{e}^{j}$ or $X_{i}^{j}$ of j-th neuron in *e* or *i* group) obeyed the non-homogeneous Poisson process.

(Equation 5) $$X_{e,i}^{j}\left(t\right)=\sum_{\left\langle t_{l}\right\rangle }\delta_{e,i}^{j}\left(t-t_{i}\right)$$

Unit impulse function represented that a neuron fired at time $t_{i}$ when the membrane potential *u>h*.

Excitatory membrane potentials $u_{e}^{j}\left(t\right)$ and inhibitory membrane potentials $u_{i}^{j}\left(t\right)$ were set as follows:

(Equation 6) $$\alpha_{e}^{-1}\frac{du_{e}^{j}\left(t\right)}{dt}=u_{e}^{j}\left(t\right)+bv_{e}^{j}\left(t\right)+G_{ee}^{j}\left(t\right)+G_{ie}^{j}\left(t\right)+\sqrt{2D}\varphi_{e}^{j}\left(t\right)\;j=1,\ldots ,N_{e}$$

(Equation 7) $$\alpha_{i}^{-1}\frac{du_{e}^{j}\left(t\right)}{dt}=u_{i}^{j}\left(t\right)+bv_{i}^{j}\left(t\right)+G_{ei}^{j}\left(t\right)+G_{ii}^{j}\left(t\right)+\sqrt{2D}\varphi_{i}^{j}\left(t\right)\;j=1,\ldots ,N_{i}$$

(Equation 8) $$a^{-1}\frac{dv_{e}^{j}\left(t\right)}{dt}={-v}_{e}^{j}\left(t\right)+u_{e}^{j}\left(t\right)\;j=1,\ldots ,N_{e}$$

(Equation 9) $$a^{-1}\frac{dv_{i}^{j}\left(t\right)}{dt}={-v}_{i}^{j}\left(t\right)+u_{i}^{j}\left(t\right)\;j=1,\ldots ,N_{i}$$

Where

(Equation 10) $$G_{ee}^{j}\left(t\right)=\sum_{k=1}^{Ne}W_{ee}^{jk}\left(c\right)\cdot {EPSP}^{k}\left(t-\tau^{jk}\right)$$

(Equation 11) $$G_{ie}^{j}\left(t\right)=\sum_{k=1}^{Ni}W_{ie}^{jk}\left(c\right)\cdot {IPSP}^{k}\left(t-\tau^{jk}\right)$$

(Equation 12) $$G_{ei}^{j}\left(t\right)=\sum_{k=1}^{Ne}W_{ei}^{jk}\left(c\right)\cdot {EPSP}^{k}\left(t-\tau^{jk}\right)$$

(Equation 13) $$G_{ii}^{j}\left(t\right)=\sum_{k=1}^{Ni}W_{ii}^{jk}\left(c\right)\cdot {IPSP}^{k}\left(t-\tau^{jk}\right)$$

Here, $N_{e}$ and $N_{i}$ represented the number of excitatory neurons and inhibitory neurons, respectively, and ${EPSP}^{k}\left(t\right)$ and ${IPSP}^{k}\left(t\right)$ showed afferent postsynaptic excitatory and inhibitory potentials with parameter $\tau_{m}$:

(Equation 14) $${EPSP}^{k}\left(t\right)=\int_{0}^{t}X_{e}^{j}\left(s\right)\frac{1}{\tau_{m}}e^{-\left(t-s\right)/\tau_{m}}ds$$

(Equation 15) $${IPSP}^{k}\left(t\right)=\int_{0}^{t}X_{i}^{j}\left(s\right)\frac{1}{\tau_{m}}e^{-\left(t-s\right)/\tau_{m}}ds$$

and synaptic weights:

(Equation 16) $$W_{nm}^{jk}\left(c\right)=w_{nm}^{o}\left(c\right)\exp\left[-\sigma_{nm}^{2}\left|x_{n}\left(j\right)-x_{m}\left(k\right)\right|\right]$$

Where *n*, *m* represented *e* or *i,* denoting the excitatory weight or the inhibitory weight, respectively. All *e* neurons were uniformly arranged on a one-dimensional space with length ω, and all *i* neurons were uniformly arranged on another one-dimensional spatial domain with length ω too. The position of the neuron was defined as the distance between the neuron and the left side of its space. $x_{n}\left(j\right)$ and $x_{m}\left(k\right)$ referred to the position of *j*-th *n*-type neuron and *k*-th *m*-type neuron. If *n* represented *e*, $\sigma_{nm}^{2}=\sigma_{e}^{2}$; while if *n* represented *i*, $\sigma_{nm}^{2}=\sigma_{i}^{2}$. Synaptic weights had the probability of 1-*c* to be set to 0 in order to reflect the sparseness of the connection. The structure of the network was shown in Figure 7A. $w_{ie}^{o}\left(c\right)=$ $w_{ii}^{o}\left(c\right)=w_{i}^{o}\left(c\right)$ was set ten times as large as $w_{ee}^{o}\left(c\right)=w_{ei}^{o}\left(c\right)=w_{e}^{o}\left(c\right)$ in the model based on the previous evidence about the EPSP/IPSP ratio of nearly 0.1.^69^

The network neural activity potential output was calculated as the mean potential among all *e* neurons’ potential and *i* neurons’ potential. The parameter value in the model was shown in the below table.

**Table:** model parameter

Parameter	Value	Parameter	Value
ω	10	*b*	0.01
$N_{e}$	800	*c*	0.8
$N_{i}$	200	$w_{e}^{o}$	35
*h*	0	$w_{i}^{o}$	−350
$\tau_{m}$	10 ms	$\sigma_{e}^{2}$	1.0
$\alpha_{e}$	1.0	$\sigma_{i}^{2}$	0.5
$\alpha_{i}$	1.5	*D*	0.001
*a*	0.01	*dt*	1 ms

### Quantification and statistical analysis

Whenever only two conditions were compared, two-sides paired t-test was applied. And Cohen’s d effect size^70^ was reported for each comparison. For the topography significance of the fE/I ratio change, Bonferroni corrections were conducted among all electrodes to control the false significant rate. The number of subjects ***n*** can be found in the figures and figure legends. The results of statistic can be found in the section of results, reporting the mean, standard deviation, p value and Cohen’s d effect size. The statistics are implemented with the function *ttest(*) in MATLAB R2018a. All significances were judged as P-value <0.05.

## Data Availability

Data has been deposited at figshare and is publically available as of the date of publication. The link is listed in the key resources table. All original code has been deposited at figshare and is publically available as of the date of publication. The link is listed in the key resources table. Any additional information required to reanalyze the data reported in this paper is available from the lead contact upon request.
