# Supplementary material for: Natural brain state change with E/I balance shifting toward inhibition is associated with vigilance impairment
Source: iScience. 2023 Sep 20;26(10):107963. doi: 10.1016/j.isci.2023.107963 (PMC10562778; doi:10.1016/j.isci.2023.107963)
Supplement: Document S1. Figures S1–S4 [file mmc1.pdf]

iScience, Volume *26*

Supplemental information

Natural brain state change with E/I balance shifting toward inhibition is associated with vigilance impairment

Binghao Yang, Haoran Zhang, Tianzi Jiang, and Shan Yu

Supplemental information

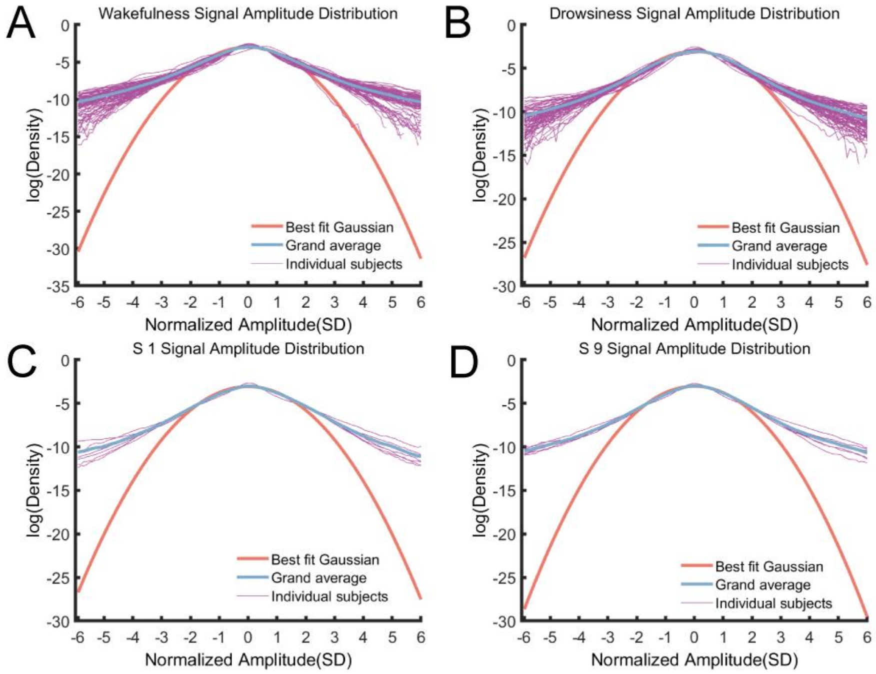

Figure S1, Threshold determination, related to Figure 3 and Figure 5. (A) Wakefulness of the postprandial somnolence group. (B) Drowsiness of the postprandial somnolence group. (C) Wakefulness of the sleep deprivation group. (D) Drowsiness of the sleep deprivation group. The purple thin curve represents the z-normalized signal amplitude distribution of the single subject. The blue thick curve represents the grand average of distributions across all subjects. The red thick curve represents the best fit Gaussian distribution. Note that the grand average and the Gaussian fit start deviating from one another at around ± 2.5 SD among all four panels.

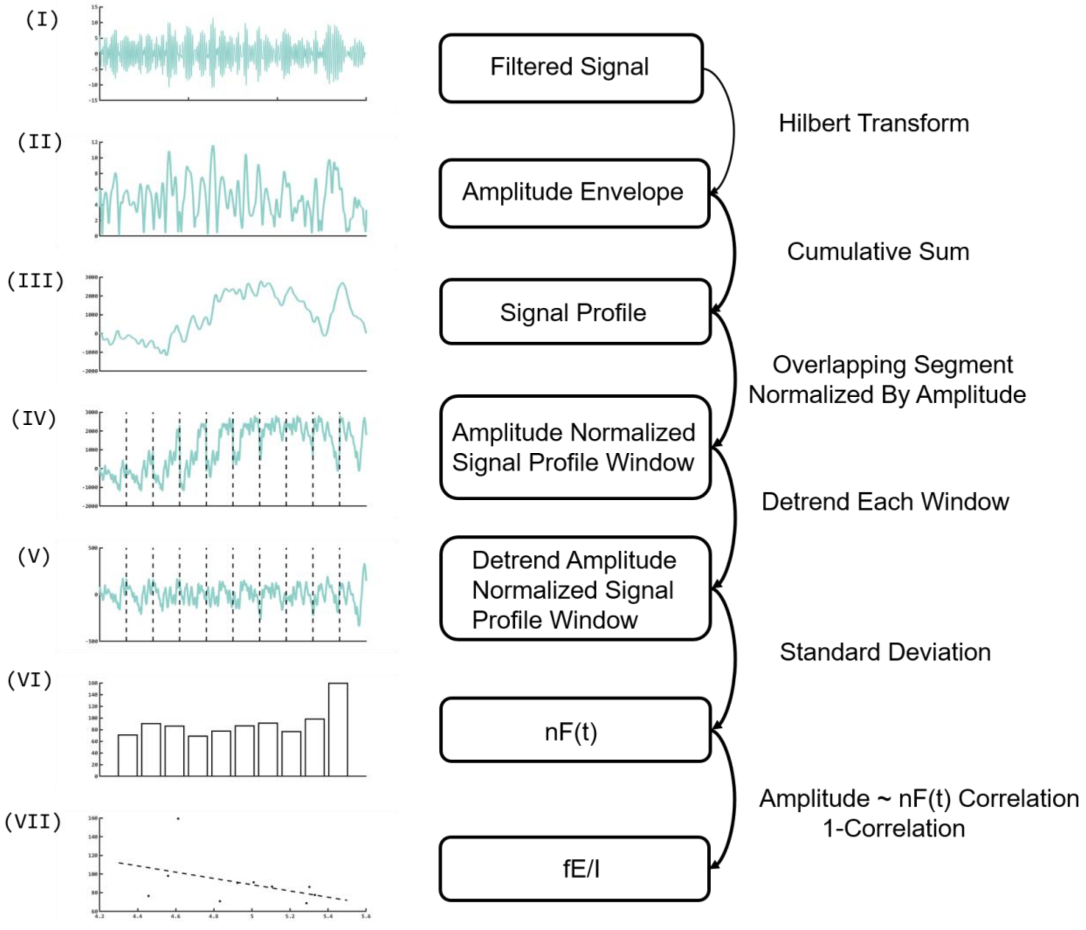

Figure S2 The fE/I calculation procedure, related to Figure 4 and Figure 5.

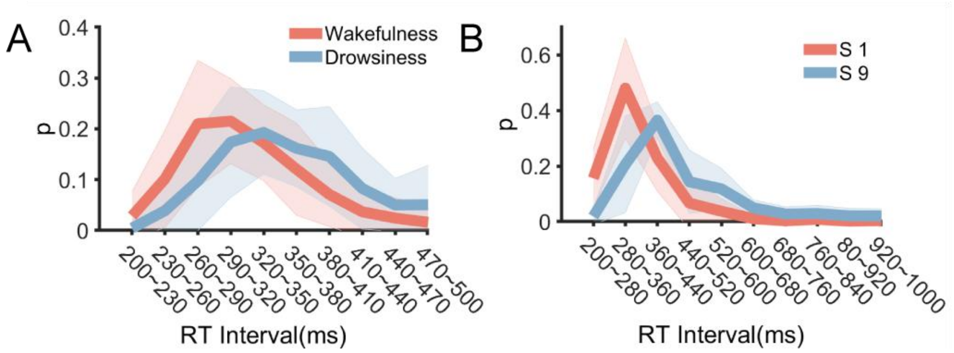

Figure S3 RT distribution, related to Figure 2. (A) RT distribution of the wakefulness (red curve) and the postprandial somnolence (blue curve). (B) RT distribution of the wakefulness (red curve) and the sleep deprivation (blue curve). The solid line represents the mean value, and the shadow represents the standard deviation. Note that the RT distribution of the postprandial somnolence or the sleep deprivation shifts towards a higher value compared with the wakefulness condition.

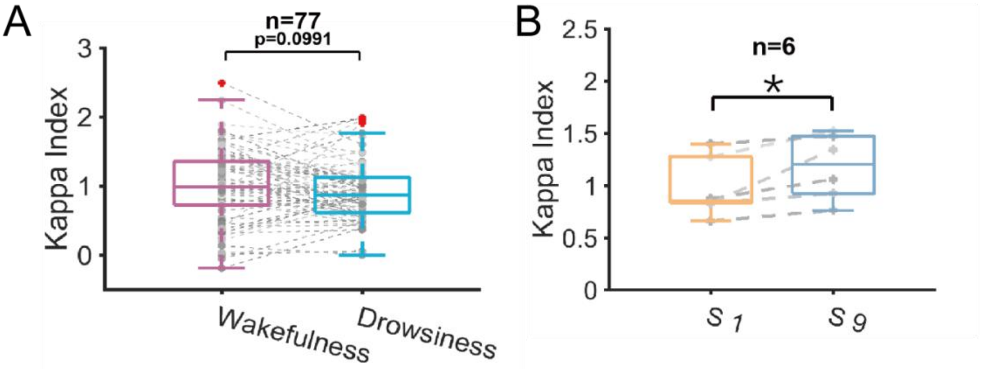

Figure S4 Analysis of kappa index, related to Figure 3 and Figure 5. (A) Changes in the kappa index of the postprandial somnolence group (n=77). (B) Changes in the kappa index of the sleep deprivation group (n=6).
